# Health-related quality of life by type of breast surgery in women with primary breast cancer: prospective longitudinal cohort study

**DOI:** 10.1093/bjsopen/zrae042

**Published:** 2024-06-03

**Authors:** Kim Gulis, Julia Ellbrant, Pär-Ola Bendahl, Tor Svensjö, Lisa Rydén

**Affiliations:** Department of Surgery, Kristianstad Central Hospital, Kristianstad, Sweden; Department of Clinical Sciences Lund, Division of Surgery, Lund University, Lund, Sweden; Department of Clinical Sciences Lund, Division of Surgery, Lund University, Lund, Sweden; Department of Surgery, Skåne University Hospital, Malmö, Sweden; Department of Clinical Sciences Lund, Division of Oncology, Lund University, Lund, Sweden; Department of Surgery, Kristianstad Central Hospital, Kristianstad, Sweden; Department of Clinical Sciences Lund, Division of Surgery, Lund University, Lund, Sweden; Department of Surgery, Skåne University Hospital, Malmö, Sweden

## Abstract

**Background:**

Health-related quality of life and patient-related outcome measures for patients with cancer have gained increased interest over the last decade. However, few prospective studies with longitudinal data evaluated health-related quality of life in patients with breast cancer. This study aimed to investigate how health-related quality of life changed from the time of diagnosis to 1 year after breast cancer surgery for the main surgical techniques.

**Methods:**

This prospective longitudinal single-centre study included patients with primary breast cancer diagnosed in 2019–2020 who underwent surgery. Patients completed a health-related quality of life questionnaire (Breast-Q) at baseline. One year after surgery, they completed the Breast-Q a second time, the EORTC (European Organization for Research and Treatment of Cancer) quality of life questionnaire-C30 and the quality of life questionnaire-BR23. Analysis of variance and Kruskal–Wallis tests were used to evaluate the differences in health-related quality of life between surgical groups. Analysis of covariance with robust standard errors was used to adjust for confounders.

**Results:**

In total, 340 patients were included in the study; 160 patients received oncoplastic partial mastectomy, 112 received partial mastectomy, 42 received mastectomy and 26 had mastectomy with immediate reconstruction. Patients that had partial mastectomy or oncoplastic partial mastectomy were more satisfied with their breasts (*P* < 0.001), had a better body image (*P* = 0.006) and higher sexual functioning scores (*P* = 0.027) than patients who had a mastectomy with/without reconstruction. The oncoplastic and mastectomy with reconstruction groups had more breast symptoms than other groups (*P* < 0.001), and the mastectomy group had the least symptoms from the chest area.

**Conclusion:**

Partial mastectomy and oncoplastic partial mastectomy have the best outcomes in terms of breast satisfaction, body image and sexual functioning. This highlights the importance of preserving the breast when feasible and underscores that breast reconstruction is not equal to breast conservation.

**Registration number:** NCT04227613 (http://www.clinicaltrials.gov).

## Teaser

This prospective longitudinal observational study presents comprehensive data on health-related quality of life in patients with breast cancer. This study found that patients who had BCS or OPBS were more satisfied with their breasts and had a better body image after surgery compared with those that received mastectomy or mastectomy with immediate breast reconstruction.

## Introduction

Breast cancer affects over 8000 women in Sweden every year and is the most common type of cancer in women^1^. In 2020, 2.3 million women were diagnosed with breast cancer globally and there were 685 000 breast cancer deaths^2^. However, the breast cancer prognosis has improved substantially over recent decades because of early diagnosis and multimodal therapy^3^. Observational studies have repeatedly shown that women who are treated with breast-conserving surgery (BCS) and radiotherapy have the same survival rate as patients treated with mastectomy^4,5^. More recent studies suggested a small increase in survival in favour of BCS with radiation compared with mastectomy^6,7^.

Applying oncoplastic breast surgery (OPBS) in BCS allows a more generous resection of breast tissue, which reduces the rate of positive margins and increases patients’ satisfaction with the cosmetic outcome^8^. A Cochrane review from 2021 showed that oncoplastic surgery was not inferior to standard BCS in terms of oncological and patient-reported outcomes^9^. The different surgical techniques for OPBS follow three main paths: volume displacement, meaning redistribution of breast tissue as in mastopexy and lateral mammoplasty, breast reduction techniques such as therapeutic mammoplasty, and volume replacement (for example local perforator flaps). These oncoplastic techniques make it possible to resect larger tumours and allow patients who traditionally required mastectomy to be candidates for BCS.

With improved survival in breast cancer, interest in patient-reported outcome measures (PROMs), which quantify aspects of health-related quality of life (HRQoL) from patients’ perspectives, has increased. PROMs complement traditional oncological outcomes and offer important information regarding the patient’s views of the impact and outcomes of their surgery and treatment. Given the ongoing development of surgical techniques to preserve the breast, it is important to evaluate outcomes using PROMs, preferably with longitudinal data. However, there is a lack of studies focused on longitudinal outcomes of HRQoL following breast cancer surgery.

The Breast-Q was developed in accordance with international guidelines for instrument development and is a validated, patient-reported outcome questionnaire that can provide essential information about the impact and effectiveness of breast surgery from the patient’s perspective^10,11^. The Breast-Q modules, both pre- and postoperative, have been translated into Swedish and can be used in Sweden without language barriers.

The present prospective longitudinal single-centre study aimed to investigate changes in the HRQoL of patients with breast cancer from baseline to 1-year post breast cancer surgery. The data was examined to assess if HRQoL scores differed between surgical techniques, and postoperative scores were compared between the surgical groups. Second, this study aimed to evaluate other patient-related variables that are potentially associated with quality of life after a breast cancer diagnosis; for example, age, body mass index, smoking and psychiatric history before diagnosis.

## Methods

This study was conducted prospectively. Patients were included at the time of diagnosis with breast cancer at Kristianstad Central Hospital, Sweden. The study started in January 2019 and aimed to include at least 300 patients. Recruitment stopped on 31 December 2020 once this target had been achieved. The cohort was followed until 31 December 2021 to ensure 1-year follow-up data were captured for all patients. This study was registered at clinicaltrials.gov (Protocol ID: NCT04227613). The study was conducted in accordance with the Declaration of Helsinki and national regulations. The ethics application was approved by the Regional Ethics Review Board at Lund University, Sweden (Diary number: 2018/827).

Kristianstad Central Hospital is a tertiary hospital with an annual case load of about 300 patients with breast cancer. All patients had an outpatient visit with a surgeon at the time of diagnosis, when the first study questionnaires were distributed and answered. Following that, a second visit was conducted before surgery and a third visit around 2 weeks after surgery. The next scheduled visit was the 1-year follow-up, at which point the follow-up questionnaires were distributed and answered. This study included women with primary breast cancer diagnosed in Kristianstad Central Hospital from 1 January 2019 to 31 December 2020 who underwent surgery. Exclusion criteria were patients who lacked the ability to read and write Swedish, patients who could not understand the information provided and patients receiving palliative care. Core needle biopsy was used for preoperative diagnosis, and information about the study-specific variables (pre-, peri- and postoperative) was retrieved from each patient’s medical records (Melior™). All collected data were pseudonymized and compiled in a REDCap database.

Participating patients completed a HRQoL questionnaire at baseline (Breast-Q for BCS, mastectomy or reconstruction, as appropriate), which was defined at the time of diagnosis. They completed the Breast-Q a second time 1-year postsurgery, along with the European Organization for Research and Treatment of Cancer’s quality of life questionnaire (EORTC QLQ-C30 and QLQ-BR23 instruments).

Breast-Q scores range from 0 to 100, with a higher score indicating a better outcome. The scores are divided into modules, and each module has its own overall score. Missing values up to 50% of a module were replaced with the mean value for the answered questions in that module; if more than 50% of data for a module were missing, the overall score for that module was excluded, based on the Breast-Q user’s guide^12^. The scores within EORTC-QLQ questionnaires also range from 0 to 100, but these scales are divided into functional and symptom scales. For the functional scales, 100 is the best value, whereas 0 is the best value for the symptom scales (indicating no symptoms). QLQ-C30 addresses general HRQoL while QLQ-BR23 is breast cancer-specific HRQoL.

Patients were stratified into four groups based on the final type of surgery: oncoplastic partial mastectomy (OPBS), partial mastectomy (PME), mastectomy and mastectomy with reconstruction. All mastectomies performed for reconstruction purposes utilized nipple-sparing techniques, with subsequent immediate, implant-based reconstructions. OPBS was defined using the NOMESCO (Nordic Medico-Statistical Committee) classification of surgical procedures^13^ as all operations coded ZZR70 (flap of glandular tissue, volume displacement), ZZR05 (flap of skin and fascia, volume replacement; LiCAP or MiCAP) or HAD30/35 (breast reduction). Symmetrizing surgery was performed at the time of primary surgery when deemed necessary by the surgeon and/or the patient. Reduction mammoplasty was always done as a bilateral procedure. All operation records were reassessed to minimize the risk of errors in surgical coding.

### Statistical analyses

Changes in Breast-Q score from baseline to the 1-year follow-up were analysed with one-way analysis of variance (ANOVA), with each patient being their own control from baseline. The postoperative Breast-Q and QLQ scores for the four surgical techniques were analysed with Kruskal–Wallis tests, and analysis of covariance (ANCOVA) was used to adjust for confounders. Robust standard errors were used in the ANCOVA analyses to account for heteroscedasticity and non-normality of residuals. The score distributions by type of surgery are shown in boxplots. The four final surgical method groups were also dichotomized into BCS *versus* mastectomy, which simplified the statistical comparisons of outcomes to independent samples *t* tests. Explorative subgroup analyses with Kruskal–Wallis tests were performed to investigate differences in outcomes between the oncoplastic surgical techniques. QLQ-C30 scores were compared with norm values for a Swedish cohort^14^ and visualized with spider plots.

Breast-Q scores were compared with norm values for an American cohort because Swedish normative data for the Breast-Q were lacking^15^. Characteristics of the patients included in this study cohort were also compared with those retrieved from the Swedish national quality registry for patients with breast cancer from the same hospital and interval to assess the representativeness of this cohort. All *P* values presented were unadjusted and should be interpreted as evidence against the null hypothesis of no association, without reference to a threshold for significance.

## Results

In total, 475 patients had primary surgery during the study inclusion interval, of which 68 did not meet the inclusion criteria, 62 declined to participate and five were not invited to participate, which gave 340 patients in the final study cohort (*Fig. S1*). Overall, 160 patients received OPBS, 112 received PME, 42 received mastectomy and 26 had mastectomy with immediate reconstruction. Fifty-five patients received neoadjuvant treatment. Thirty-seven had a pre-existing psychiatric diagnosis. All patients attended the 1-year follow-up, except one patient who died during the first year after surgery. Five patients did not complete all forms and 73 patients (21.5%) chose not to answer the sexually related questions. The response rates for the other modules were around 95%. Baseline characteristics for the study cohort are shown in *Table 1*. The re-operation on frequency within the first year ranged from 2.7% (3 of 112) in the PME group (all due to non-radicality) to 31% (8 of 26) in the mastectomy with reconstruction group (six due to bleeding).

**Table 1:** Baseline patient and tumour characteristics for the 340 women in the study cohort

	Oncoplastic surgery (*n* = 160)	Partial mastectomy (*n* = 112)	Mastectomy (*n* = 42)	Mastectomy + reconstruction (*n* = 26)
**Baseline characteristics**				
Age (years), median (range)	63 (26–84)	66 (27–92)	74 (40–86)	54 (37–68)
BMI (kg/m^2^), median (range)	27 (18–41)	27 (17–40)	23 (17–38)	26 (16–46)
Breast volume (ml), median (range)	650 (125–1900)	750 (125–1800)	425 (125–1500)	650 (200–1300)
Screening detected	191 (63.1)	72 (64)	7 (16.7)	15 (57.7)
Palpable	99 (61.9)	52 (46)	36 (85.7)	14 (53.8)
Mammographic size (mm), median (range)	22 (4–70)	14 (3–45)	30 (8–90)	30 (12–130)
Ultrasound size (mm), median (range)	19 (5–60)	12 (5–42)	28 (8–100)	23.5 (7–70)
Neoadjuvant treatment	29 (18.1)	14 (12.5)	5 (11.9)	7 (26.9)
**Tumour characteristics**				
*In situ*	12 (7.5)	11 (9.8)	4 (9.5)	3 (11.5)
Ductal invasive	107 (66.9)	88 (78.6)	21 (50)	15 (57.7)
Lobular invasive	21 (13.1)	5 (4.5)	12 (28.6)	3 (11.5)
Other invasive	12 (7.5)	5 (4.5)	5 (11.9)	4 (15.4)
PCR*	8 (5)	3 (2.7)	0	1 (3.8)
**Surgical characteristics**				
Duration of primary surgery (mins), median (range)	94 (39–174)	62 (27–152)	81 (43–156)	134 (60–248)
EPBVE, median (range)**	11.8 (2–75)	5.1 (1–22)	–	–
Re-operation on	12 (7.5)	3 (2.7)	5 (11.9)	8 (30.8)
Bleeding	5 (3.1)	0	2 (4.8)	6 (23.1)
Non-radical	6 (3.8)	3 (2.7)	2 (4.8)	0
Necrosis	1 (0.6)	0	1 (2.4)	1 (3.8)
Prophylactic surgery	0	0	0	1 (3.8)
**Nodal status**				
Sentinel node surgery	139 (86.9)	101 (90.2)	29 (69.0)	22 (84.6)
Axillary clearance	31 (19.4)	12 (10.7)	17 (40.5)	4 (15.4)
Malignant nodes	53 (35.3)	16 (15.4)	24 (63.2)	8 (33.3)
**Adjuvant therapy**				
Endocrine therapy				
No	48 (30)	40 (35.7)	12 (28.6)	12 (46.2)
5 years	71 (44.4)	61 (54.5)	10 (23.8)	9 (34.6)
>5 years	41 (25.6)	11 (9.8)	20 (47.6)	5 (19.2)
Radiotherapy	156 (97.5)	93 (83.8)	19 (45.2)	8 (30.8)
Chemotherapy	67 (41.9)	29 (25.9)	17 (40.5)	12 (46.2)
HER-2 directed therapy	21 (13.1)	12 (10.7)	6 (14.3)	2 (7.7)

Values are *n* (%) unless otherwise indicated. Missing data: breast volume (0.9%), EPBVE (estimated percentage of breast volume excised; 1.1%), lymph node status (0.3% and 6.8% never had axillary surgery), mammographic size (7.3% not visible), ultrasound size (9.4% not visible). *Pathological complete response. **Estimated percentage breast volume excised, calculated as specimen weight divided by breast volume and times 100, reported as a percentage. HER-2, human epidermal growth factor receptor 2.

**Fig. 1:**
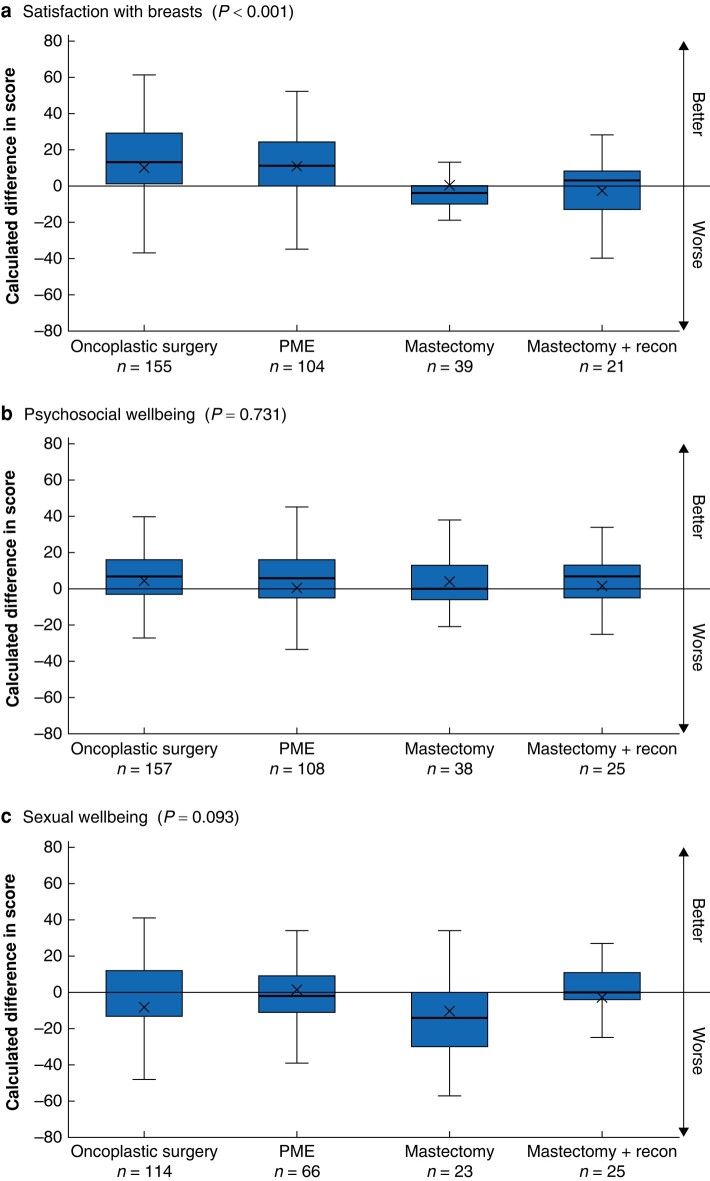
**Difference in post- and preoperative Breast-Q scores between the surgical groups, analysed with analysis of variance** **a** Breast-Q module ‘satisfaction with breast’, **b** Breast-Q module ‘psychosocial wellbeing’, **c** Breast-Q module ‘sexual wellbeing’. PME, partial mastectomy. Box, interquartile range; whiskers, minimum and maximum values excluding outliers; horizontal line, median; X, mean.

The differences in post- *versus* preoperative Breast-Q scores are shown in *Fig. 1*. The mastectomy and mastectomy with immediate reconstruction group showed lower satisfaction with their breasts compared with the PME and OPBS groups (*P* < 0.001). The satisfaction with breasts score in the OPBS and PME groups increased by an average of 13.8 and 11.9 units respectively. However, satisfaction with breasts decreased in the mastectomy and the mastectomy with reconstruction groups by 1.9 and 2.3 units respectively. The evidence for the difference in satisfaction with breasts remained strong when the surgical groups were merged to BCS *versus* mastectomy.

The other two Breast-Q modules with longitudinal data showed weak evidence for differences between the four surgical groups, although there was a trend towards lower sexual wellbeing in the mastectomy group compared with the other groups (*P* = 0.093; three degrees of freedom test). At 1-year postsurgery, the mastectomy group’s mean sexual wellbeing score had decreased by an average of 12.7 units, and that in the other groups had decreased by 1.0–2.5 units.

**Fig. 2:**
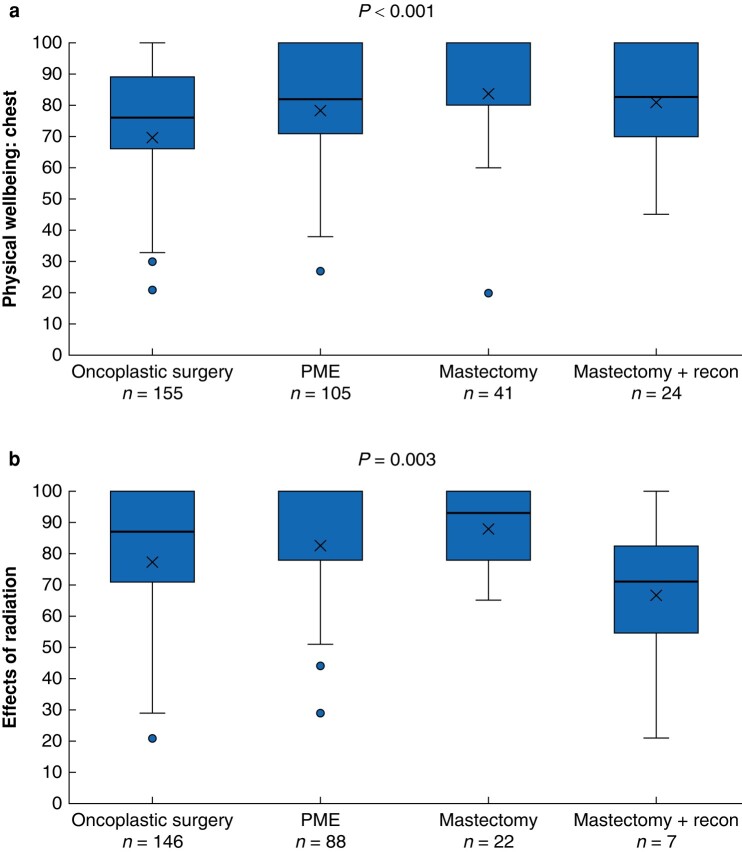
**Comparison of postoperative Breast-Q scores between the surgical groups, analysed with Kruskal–Wallis tests** **a** Breast-Q module ‘physical wellbeing: chest’, **b** Breast-Q module ‘effects of radiation’. PME, partial mastectomy. Box, interquartile range; whiskers, minimum and maximum values excluding outliers; horizontal line, median; X, mean; blue dot, outlier.

The results for two postoperative Breast-Q modules are shown in *Fig. 2*. The mastectomy group had the highest scores for physical wellbeing: chest (*P* < 0.001), with a mean score of 89. The PME and mastectomy with reconstruction groups both had a mean score of 82 and the OPBS group a mean score of 75. The module for effects of radiation (which patients only completed if they had received radiation therapy) showed the mastectomy with reconstruction group had lower scores than the other groups (*P* = 0.003), with a mean score of 72. The mean scores for the other surgical groups were: PME = 88, OPBS = 81 and mastectomy = 89.

The ANCOVA for patients’ postoperative scores was adjusted for age at diagnosis, psychiatric diagnosis, smoking, neoadjuvant treatment and ASA score, as applicable. The results of the adjusted analyses were similar to those of the unadjusted analyses regarding the level of evidence for differences between the groups in the postoperative modules. Furthermore, adjustment for baseline scores in the analyses of changes in Breast-Q scores did not change the interpretation of differences between the surgical groups. The remaining postoperative Breast-Q modules included scores for satisfaction with the information given, satisfaction with the surgeon and satisfaction with other hospital staff, all of which showed no clinically relevant differences between the groups (*Fig. S2*).

When the Breast-Q baseline scores were compared with American normative data, the study cohort had the same mean scores for sexual wellbeing (56 points), and similar scores for satisfaction with breasts (61 *versus* 68) and psychosocial wellbeing (67 *versus* 71). Comparisons between this cohort and the Swedish national quality registry from Kristianstad Central Hospital in the same time interval are shown in *Table S3*; the only relevant difference was a slightly higher proportion of patients with malignant lymph nodes in the study cohort (29.7%, 101 of 340) compared with the registry data (26.6%, 106 of 398), suggesting that patients in this study had slightly more advanced disease than excluded patients.

**Fig. 3:**
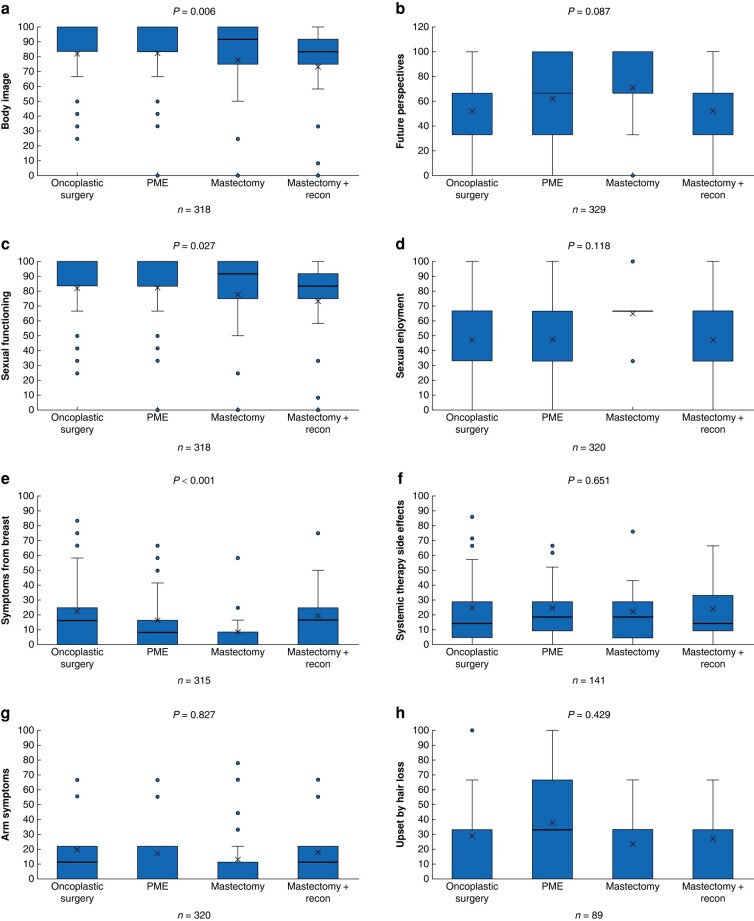
**Comparison of postoperative QLQ-BR23 between the surgical groups, analysed with Kruskal–Wallis tests** **a** Body image, **b** Future perspectives, **c** Sexual functioning, **d** Sexual enjoyment, **e** Symptoms from breast, **f** Systemic therapy side effects, **g** Arm symptoms, **h** Upset by hair loss. PME, partial mastectomy. Box, interquartile range; whiskers, minimum and maximum values excluding outliers; horizontal line, median; X, mean; blue dot, outlier.

**Fig. 4:**
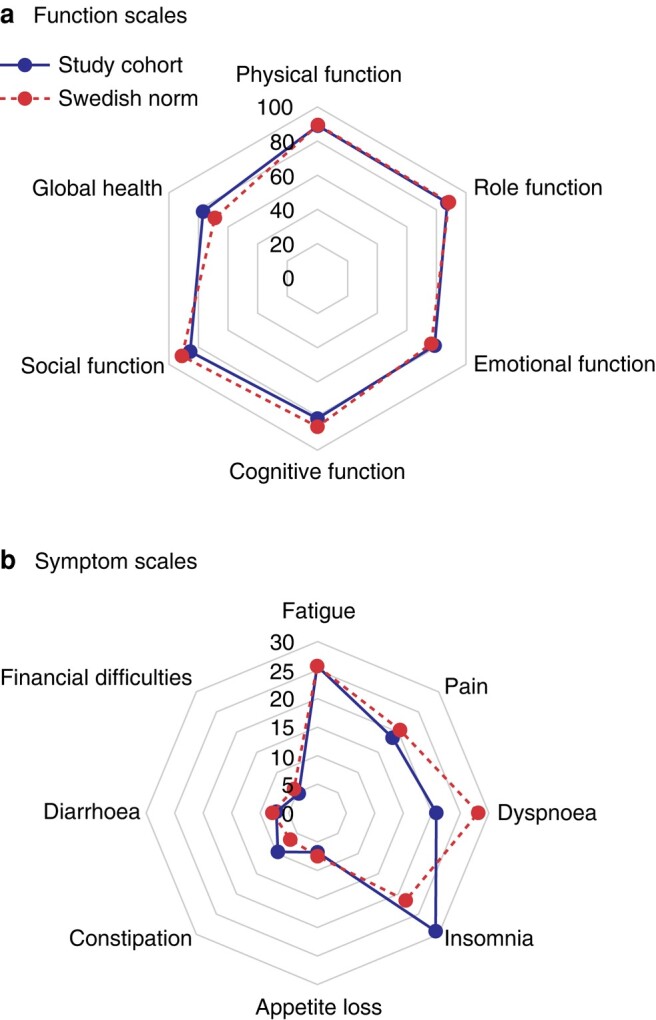
**Comparison of postoperative mean scores for quality of life questionnaire (QLQ)-C30 in the study cohort and norm values for QLQ-C30** **a** Function scales, **b** Symptom scales.

The QLQ-BR23 postoperative scores are shown in *Fig. 3*. The mastectomy and reconstruction group had lower mean scores for body image and sexual functioning (77 and 69 respectively) compared with the other groups. On average, the breast conserving groups scored above 80 in both body image (*P* = 0.027) and sexual functioning (*P* = 0.006). The mastectomy group also had the fewest symptoms from the breast (mean score 6.5), followed by the PME group (mean score 12), compared with the oncoplastic and mastectomy with reconstruction groups (both with a mean score of 17) (*P* < 0.001). The other scales showed no relevant differences between the groups. The evidence was generally weak for differences in QLQ-C30 scores between the groups (*Fig. S4*). The mean QLQ-C30 score for the whole study cohort was compared with norm values for a Swedish cohort (*Fig. 4*), which showed this cohort had slightly lower mean scores for cognitive function and social function and higher insomnia and constipation scores than the Swedish cohort, but no difference/better scores in all other subscales. Interestingly, the global health mean score was 7.7 points higher in the study group than in the reference cohort.

## Discussion

This prospective longitudinal observational study presents comprehensive data on HRQoL in patients with breast cancer. According to a recently accepted meta-analysis on Breast-Q, there has been no previous prospective study comparing HRQoL of BCS and OPBS of a comparable size to this study^16^.

The longitudinal design allowed each patient to serve as their own control, thereby enhancing the study's statistical power to discern differences between groups. Additionally, this study featured a substantial cohort size with a minimal proportion lost to follow-up and negligible missing values, further strengthening the reliability and validity of its findings. This study found that patients who had BCS or OPBS were more satisfied with their breasts and had a better body image after surgery compared with those that received mastectomy or mastectomy with immediate breast reconstruction. More breast symptoms were observed in the OPBS and mastectomy with reconstruction groups. Interestingly, the group with the fewest symptoms from the chest area was the mastectomy group. This could be explained by the fact that many patients who undergo mastectomy do not receive postoperative radiotherapy, the exception being those with malignant lymph nodes.

The Breast-Q questionnaire was developed in 2007 and to date, around 1000 publications have included Breast-Q data. A systematic review published in 2019 evaluated OPBS and HRQoL, in which six studies met the inclusion criteria. Only one of the included studies provided data in favour of OPBS, which was a study from 2010 that used a different HRQoL questionnaire than the Breast-Q^17^. The authors of the systematic review strongly recommended further cohort studies on HRQoL with OPBS, serving as an incentive for the present study.

Another recent scoping review of the application of the Breast-Q highlighted a need for prospective collection of centre-specific data for every type of breast surgery, which was analogous to the present study design^18^. The evaluation of OPBS compared with traditional techniques in terms of oncological outcomes and from patients’ perspectives is needed to determine if more extensive surgical choices are of value for patients. Baseline data in such studies is necessary to evaluate HRQoL and satisfaction changes for individual patients.

In 2013, a prospective trial was published that evaluated HRQoL in patients with BCS with radiotherapy^19^; however, that study solely used the QLQ-C30, which measures HRQoL generally and was not specifically developed for breast cancer. A case-control study from 2016 examined HRQoL using the Breast-Q in different surgical techniques^20^. In that study, data were collected retrospectively, and the outcome showed lower Breast-Q scores for BCS compared with mastectomy with reconstruction, and patients reported asymmetry after BCS as a major issue. However, the study interval was 2009–2013, which suggested that newer OPBS techniques may not have been used^20^. Two Swedish studies have evaluated the Breast-Q after surgery. Dahlbäck *et al.* retrospectively distributed the Breast-Q questionnaire (median 5 years after surgery) to 489 (of which 349 answered) patients that received BCS, and HRQoL was compared with cosmetic outcomes^21^. They found an association between low scores in cosmetic outcomes and lower scores in the Breast-Q; there was no comparison to a mastectomy group or with OPBS. The second study involving a retrospective assessment of unilateral OPBS was published in 2019 and found that the mean score for satisfaction with breasts was 74 (which was consistent with this study’s OPBS postoperative mean score of 73), and 11% wanted a contralateral surgery^22^. In addition, consistent with the current data, another retrospective study published in 2019 used the Breast-Q to compare BCS with mastectomy with implant reconstruction and concluded that breast satisfaction and HRQoL scores were highest for BCS^23^. A Danish retrospective study compared BCS with OPBS using the Breast-Q and showed better HRQoL outcomes for the OPBS group^24^. Stolpner *et al.* reported the results of a prospective study conducted in 2021 that evaluated BCS using the Breast-Q score and was intended as a benchmark for further studies; their mean score for satisfaction with breasts was lower than that in the present study cohort (67 *versus* 71)^25^.

A recent cross-sectional study compared OPBS levels 1 and 2 using the Breast-Q and found no differences in Breast-Q postoperative scores^26^. However, that study invited 968 patients but only 232 questionnaires were returned. Another study from 2022 explored postoperative Breast-Q for different OPBS techniques and showed high scores for all modules compared with the same normative values for healthy women, which were used in the present study^27^. A cross-sectional study from 2023 examined differences in patient-reported outcomes between OPBS and PME using the Breast-Q and a questionnaire called DASH (Diseases of the Arm, Shoulder, and Hand) and found no differences in median values at a group level^28^. This short summary shows the lack of prospective studies with baseline data that investigated the effects of different surgical techniques on HRQoL in patients with breast cancer.

The strengths of this study included the prospective longitudinal data collection; each patient was their own control, which increased the power to detect differences between groups. Another strength was the relatively large cohort with few losses to follow-up and few missing values. A limitation of this study was that it was a single-centre study. Therefore, comparison with data from other centres for validation would be of value. Second, only 1-year follow-up data was available; however, a 5-year follow-up is scheduled, and will be ready for analysis by the end of 2025. Furthermore, data on cosmetic outcomes were collected for the same cohort and will be evaluated along with HRQoL data. This study highlighted the importance of surgeons trained in OPBS techniques in breast cancer care by showing better HRQoL outcomes for the BCS group. Adjustment for known confounders did not change the interpretation of the results, showing the results were robust.

BCS and OPBS seem to impact HRQoL less compared with mastectomy with or without reconstruction. Patient selection is vital in breast cancer surgery, and it is important to choose the optimal surgery for each patient. This is highlighted by the fact that patients who had more extensive surgery reported more breast-related symptoms after surgery. There is a need for further prospective longitudinal studies to confirm the present findings. Preservation of the breast should always be the first choice in breast cancer surgery, and OPBS gives patients who previously would have been at risk of mastectomy the opportunity to receive BCS instead. With increasing survival, patients will often have to live with the outcomes of surgery for a long interval of time.

## Supplementary Material

zrae042_Supplementary_Data

## Data Availability

The data set used in this study may be available from the corresponding author upon reasonable request.
