# Supplementary material for: Health-related quality of life by type of breast surgery in women with primary breast cancer: prospective longitudinal cohort study
Source: BJS Open. 2024 Jun 3;8(3):zrae042. doi: 10.1093/bjsopen/zrae042 (PMC11146426; doi:10.1093/bjsopen/zrae042)
Supplement: zrae042_Supplementary_Data [file zrae042_supplementary_data.docx]

**A prospective longitudinal cohort study of health-related quality of life by type of breast surgery in women with primary breast cancer**

Gulis Kim^1,2^, Ellbrant Julia^2,3^, Bendahl Pär-Ola^4^, Svensjö Tor^1*^, Rydén Lisa^2,3*^

^1^ Department of Surgery, Kristianstad Central Hospital, Kristianstad, Sweden.

^2^ Department of Clinical Sciences Lund, Division of Surgery, Lund University, Lund, Sweden.

^3^ Department of Surgery, Skåne University Hospital, Malmö, Sweden.

^4^ Department of Clinical Sciences Lund, Division of Oncology, Lund University, Lund, Sweden.

*These authors shared the last authorship

**Corresponding Author:**

**Kim Gulis**Department of Surgery
J A Hedlunds väg 5
Kristianstad Central Hospital
SE-29133 Kristianstad, Sweden
Email: Kim.Gulis@med.lu.se
Telephone: 0046 44 309 22 86

ORCID ID: 0000-0002-4089-0760

**Supplementary Materials - Index**

| **Supplementary Figures and Tables** |  |
| --- | --- |
| Figure S1 | *pag. 2* |
| Figure S2 | *pag. 3* |
| Table S3  Figure S4 | *pag. 3*  *pag. 4* |
|  |  |

Figure S1. Flowchart of the study

Figure S2 Postoperative Breast-Q scores

Breast-Q Satisfaction with information from surgeon


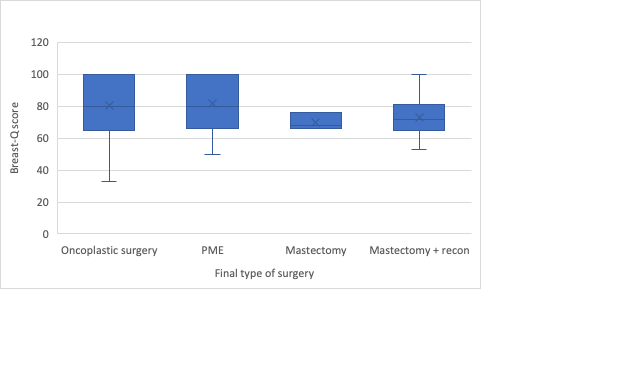


N=273

P=0.166 (Kruskal-Wallis test)

Breast-Q Satisfaction with surgeon

N=326

P=0.498 (Kruskal-Wallis test)

Breast-Q Surgical team

N=320

P=0.138 (Kruskal-Wallis test)

Breast-Q Other members of staff

N=321

P=0.432 (Kruskal-Wallis test)

Table S3. Comparison to Swedish national quality registry for breast cancer

Inclusion period 2019-2020 for patients diagnosed at Kristianstad hospital

|  | **Swedish national quality registry** (n=541) | **Study cohort** (n=340) |
| --- | --- | --- |
| **Age, years** | 66 | 64 |
| **Tumour size (postoperative), mm** | 14 mm | 14 mm |
| **Malignant lymph nodes, %** | 26.6 | 29.7% |

Figure S4. QLQ C30 postoperative scores

**
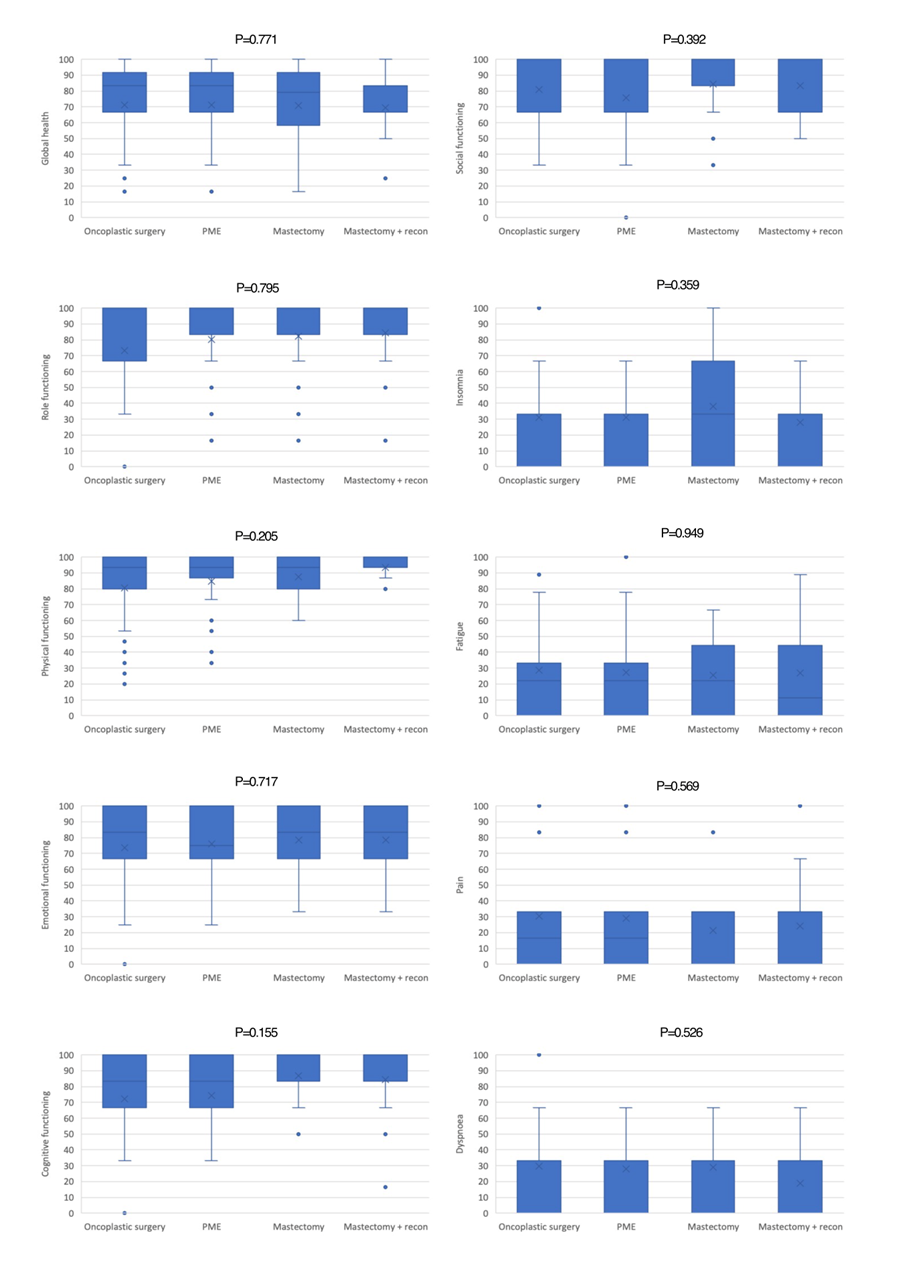
**
